# Validation of the controlled ovarian stimulation impact measure (COSI): assessing the patient perspective

**DOI:** 10.1186/1477-7525-11-130

**Published:** 2013-07-31

**Authors:** Meryl Brod, Hein Fennema

**Affiliations:** 1 The Brod Group, 219 Julia Avenue, Mill Valley, CA 94941, USA; 2 Merck Sharp & Dohme BV, Molenstraat 11, 5342 CC Oss, The Netherlands

**Keywords:** Controlled ovarian stimulation, Validation, Controlled ovarian stimulation impact measure, Quality of life

## Abstract

**Background:**

Controlled Ovarian Stimulation (COS) is the first step for in vitro fertilization (IVF) treatment, a treatment often described and experienced as stressful to patients and their partners. COS also requires concerted efforts by the patients in administering medication and general compliance to treatment protocols. Little is known about the impacts on patients that may be specific to this important first step in treatment. The absence of a conceptually sound and well-validated measure assessing patient experience and functioning during ovarian stimulation has been an obstacle to understanding the impacts of ovarian stimulation on women pursuing IVF. To address this gap, the Controlled Ovarian Stimulation Impact Measure (COSI) was developed based upon accepted methods for designing patient reported outcome (PRO) measures. The purpose of this study was to psychometrically validate the COSI.

**Methods:**

267 patients from three countries (Ireland, United Kingdom, United States) were administered the COSI. Psychometric validation was conducted according to an *a priori* statistical analysis plan.

**Results:**

The final 28-item COSI was found to have robust scale structure with four domains: Interference in Daily Life (Work and Home), Injection Burden, Psychological Health and Compliance Worry. Internal consistency of all domains was adequate (between 0.80 to 0.87) as was test-retest reliability (between 0.72-0.87). All a-priori hypotheses for convergent and known-groups validity tests were met.

**Conclusions:**

There is a measurable impact of COS on patient functioning and well-being. The COSI is a well-developed and validated PRO measure of this impact. Future work should include examination of responsiveness and confirmation of concepts in non-western countries.

## Background

In vitro fertilization (IVF) has been documented as a potentially difficult treatment process with impacts on patients and their partners. Researchers have observed increased anxiety levels in patients at several junctures within the IVF cycle [1]. These points of increased impact include prior to the beginning of a cycle [2-6]; before oocyte retrieval and prior to embryo transfer [7-10]; before administering a pregnancy test [9-11]; waiting for the results of IVF [9,11]; and following an unsuccessful IVF [2-4,12-14]. Furthermore, there is some evidence that infertility-related stress, anxiety, and depressive symptoms may negatively affect infertility treatment outcome [15-19], however there are conflicting results in this research area [20]. Despite these reports, little attention has been given to discrete procedures within the IVF treatment process and impacts on patients that may be specific to these procedures and treatment phases.

IVF treatment involves five phases: (i) ovarian stimulation; (ii) oocyte retrieval; (iii) fertilization followed by embryo culture; (iv) embryo transfer; and (v) the luteal phase. Controlled Ovarian Stimulation (COS) is the first step in IVF treatment. COS also requires concerted efforts by the patients, and perhaps their partners, in administering medication and general compliance to treatment protocols. A recent study by the first author, consisting of patient interviews and focus groups in three countries, revealed that COS can place significant burdens on patients as a discrete phase in the IVF process, impacts so great that they could be recalled up to a year after the COS [21]. Furthermore, the impacts were sustained regardless of the outcome of IVF, whether or not pregnancy was achieved [21]. This study demonstrated that the frequent injection and monitoring associated with COS contributed to the burden specific to COS, impacting psychological and daily functioning. Despite this one study, little is known about the specific extent and burden of COS impacts on women pursuing IVF as currently the only validated measure of the impact of fertility problems on quality of life is the FertiQoL. However, the FertiQoL does not specifically focus on the impact of COS and no COS treatment specific patient reported outcome measure (PRO) of the impacts has been available.

To address this gap, the Controlled Ovarian Stimulation Impact Measure (COSI) was developed in order to provide a way of assessing and understanding the impacts of controlled ovarian stimulation on functioning and well-being for patients undergoing IVF. The development process followed accepted principles of measure development for patient reported outcomes [22]. To ensure content validity of the COSI, the concept elicitation phase included literature review, expert interviews, and direct input from 47 women who have experienced COS [21]. Based on these findings, a conceptual model was developed and 46 possible items were generated from the model using the patient/participant voices. The purpose of this study was to psychometrically validate the COSI by developing a formal measurement model that identifies domains and subscales from the initial item pool, and explore the reliability and validity of the COSI using a pre-specified analysis plan [23,24].

## Methods

### Subjects

This multicenter, non-interventional study was conducted in 10 clinics located in Ireland (1 site), the United Kingdom (3 sites), and the United States (6 sites). Women receiving Controlled Ovarian Stimulation (COS) for IVF and/or intracytoplasmic sperm injection (ICSI) treatment were enrolled in the study. Ethics approval was received in all 3 countries (US: Independent Review Consulting #3239–001, UK: reference #07/H1306/161, Ireland: Rotunda Hospital, no number provided).

Eligibility criteria included women who were scheduled to undergo COS for IVF/ICSI within the next month, were between the ages of 18–39 years of age at the time of signing informed consent, able to read and speak English, and willing and able to sign informed consent. Women known to have either psychiatric illness or alcohol/drug abuse within the previous 12 months were not eligible to participate.

All eligible women who had appointments to proceed to human chorionic gonadotrophin (hCG) administration (the final COS injection) as part of their treatment were consecutively asked to participate in the study. Study participants who did not proceed to hCG administration and had a cancelled cycle were not allowed to continue in the study. These participants were dropped from the study and replaced by another patient.

The routine medical procedures and/or medications of the participants were not changed in any way by participation in the study. This study was reviewed and approved by an Institutional Review Board and all patients signed an informed consent.

### Procedure

Participants were asked to complete a battery of questionnaires at the day of hCG or the day after. Women were given the battery to take home with them at their final monitoring visit prior to hCG administration, when the decision was made to proceed to hCG injection. If the decision to proceed to hCG was expected to be made when the woman was not in the clinic, the battery was given at the participant’s monitoring visit on stimulation day 7 or 8 of their COS treatment. Women were informed that they should complete all questionnaires in the validation battery either the day of or the day after their hCG injection, without interference or discussion from another person, and return the completed battery to the clinic at the oocyte retrieval appointment.

At the embryo transfer appointment, a subsample of 178 women received the COSI retest with a pre-stamped pre-addressed envelope. They were instructed to complete the measure on day 8 following embryo transfer.

### Measures

The survey took approximately 35 minutes to complete. In order to conduct the psychometric analyses required to validate the measure, additional measures were also included in the survey battery along with the COSI. Table 1 presents which measures were employed for each of the psychometric analyses. These measures include:

**Table 1:** Hypotheses for the tests of validity

**Domain tested**	**Hypotheses**
	***Convergent Validity***
Total score	There will be a strong correlation with life satisfaction on the Q-LES-Q.
Psychological Impact	Women with greater mental health on the PGWBI will have lower domain scores.
Interference in Daily Life	Women who have higher scores on the HFRDIS will have greater domain scores.
Injection Burden	Women with greater scores on the TSQM or the ITSQ Convenience subscale will have greater domain scores.
Work	Women with greater negative scores on the Work Productivity Scale (EWPS) will have greater domain scores and women who self report a negative work impact will have lower domain scores.
Compliance Worry	Women with greater Compliance Worry will have greater treatment inconvenience.
	***Discriminate validity***
Total score	Greater fear of injection as assessed by the D-FISQ will be associated with a greater COSI score.
Psychological Impact	Women who have more supportive spouses will have greater domain scores.
Daily Life Interference	Women who work will have greater domain scores than women who do not work.
Injection Burden	Women who mix their COS medication and/or who have previous experience with self-injections will have greater Injection Burden.
Work	Women who self-report a supportive work environment will have lower domain scores.
Compliance Worry	Women who feel they were better trained to administer injections will have less Compliance Worry.

#### Diabetes fear of injecting and self-testing questionnaire (D-FISQ) - fear of self injecting (FSI) subscale

A 15-item quality-of-life subscale measuring fear of self injecting in adult diabetics. Subjects rate the items on a 4-point scale as 0 (“almost never”), 1 (“sometimes”), 2 (“often”), or 3 (“almost always”) over the past month. FSI subscale scores are calculated by summation to detect and quantify the degree of emotional, cognitive, behavioral and physiological level of fear of self-injecting [25].

#### Quality of life enjoyment and satisfaction questionnaire (Q-LES-Q) (short form)

A 16-item questionnaire assessing the degree of enjoyment and satisfaction experienced in eight areas (physical health, subjective feelings of well-being, work, household duties, school, leisure, social relationships, and general life quality). Each item is rated on a 5-point Likert scale that indicates the degree of enjoyment or satisfaction achieved during the past week (1 = “very poor”; 5 = “very good”). Scores are aggregated, with higher scores indicative of greater enjoyment or satisfaction in each domain [26].

#### Endicott work productivity scale (EWPS)

A 25-item questionnaire measuring the degree to which a medical condition affects subjects’ work functioning. Subjects rate the items on a 5-point Likert scale measuring frequency from 0 (“never”) to 4 (“almost always”) over the past week. Scores are aggregated; the maximum score possible (worst) is 100 and the best possible score is 0. Total score is based on the degree to which behaviors and subjective feelings or attitudes that are likely to reduce productivity and efficiency in work activities characterize the subject during the week before evaluation. The number of hours of work expected, the number worked, and the reason(s) why the subject worked less than usual are also collected [27].

#### Frequency, intensity, and burden of side effects rating (FIBSER)

A 3-item questionnaire measuring medication side effect impact over the past week using 3 domains: frequency, intensity, and global burden (degree interfered with day-to-day functions). Each domain is rated on a 7-point scale (Frequency: ranging from “no side effects” to “present all of the time”; Intensity: ranging from “no side effects” to “intolerable”; Burden: ranging from “no impairment” to “unable to function due to side effects”). Each specific domain is assessed as a total value and compared to develop a picture of overall effect of medication side effects, e.g., some side effects may occur infrequently, but be both intense and very burdensome to the patient or other side effects may be highly frequent, but be only of modest intensity and minimal burden [28].

#### Hot flash related daily interference scale (HFRDIS)

A 10-item questionnaire, adapted so that that it measured the degree to which ovarian stimulation interferes with nine daily activities. Subjects rate the degree of interference with each item during the previous week using a 0 (“do not interfere”) to 10 (“completely interfere”) scale. A total scale is computed by summing items. Higher scores indicate higher interference and thus, greater impact on quality of life [29].

#### Insulin treatment satisfaction questionnaire: convenience domain (ITSQ)

A 5-item domain of a 22 item questionnaire assessing treatment satisfaction for diabetic patients on insulin over the past month, adapted to assess the impact of the convenience of ovarian stimulation. All items are rated on a 7-point Likert scale and scored by transforming all items to a scale of 0–100 with the higher score (for the overall score and for each subscale) indicating better treatment satisfaction. ITSQ subscale scores are calculated by imputing the missing values based on the mean of the non-missing items [30].

#### Treatment satisfaction questionnaire for medication (TSQM)

A 14-item questionnaire measuring a patient’s satisfaction with medication. The time frame is 2–3 weeks, or since the last medication use. Items are rated on a 5- or 7-point scale according to patients’ experience with the medication in terms of satisfaction, bother/interference with side effects, ease of use and confidence [31].

#### Psychological general well-being (PGWB) index

A 22-item questionnaire measuring self-representations of intrapersonal affective or emotional states reflecting a sense of subjective well-being or distress during the past month. The PGWB includes indicators of positive and negative affective states. Questionnaire subscales are: anxiety, depressed mood, positive well-being, self control, general health and vitality. Items are rated on a 6-point scale, according to the intensity or frequency of the affective experience. Total scores on the PGWB range from 0 to 100 and are expressed as a summary score; a higher score equals better quality of life. For each item, the response option that indicates the greatest distress is scored zero; the most positive option is scored five [32].

### Statistical analysis

#### Validation strategy

Data was analyzed according to an a-priori statistical analysis plan to determine item and factor structure, assess internal and test-retest reliability, construct validity using a nomological network approach, divergent and convergent validity, discriminant validity, and to examine the minimally important difference. Validation analyses were conducted for the total COSI score as well as for each (derived) domain score. All batteries and COSI retests were double data entered into a centralized SPSS database. All analyses were performed using SAS^©^ (SAS institute, Cary).

#### Item reduction and measurement model

The preliminary version of the COSI consisted of 42 core items with 6 additional items for working women, assessing 4 core domains (Psychological, Interference in Daily Life, Convenience and Side Effects) and 1domain for Impact on Work Life. Items were rated on a 5-point Likert scale which range from either “Not at all/Never” to “Extremely/Always” with intervals (“rarely/a little”, “sometimes/somewhat”, “often/a lot”).

Analytic guidelines of the decision criteria were used to guide the process of item reduction of the preliminary 48 items. These included criteria for examination of missing data (any items having 5% or greater missing data were assessed for removal from the scale), ceiling and floor effects (if the frequency of either response extremes is greater than 50%, the item is considered to be demonstrating a ceiling or floor effect), item-rest correlations (Pearson’s correlation of .70 or greater is acceptable) and item-to-item correlations (correlation coefficients greater than 0.80 may indicate a redundancy between the items). Additionally, content validity (conceptual relevance) of items was also considered in final decisions as to whether or not an item should be deleted. This was done to ensure that important patient reported key concepts remained in the measure. Thus, final item reduction decisions were treated as an iterative process between these psychometric methods and the conceptual framework developed in the measure development process.

Factor analysis using VARIMAX rotation was performed on the correlation matrices derived from the core items comprising the COSI. In order to include all respondents, work related items were not included in the factor analysis. The final solution for factors was also rotated using oblique rotation (OBLIMIN) in order to establish the degree of correlation between domains. The most appropriate number of factors to be extracted was determined using primarily (A) residual analysis, i.e., evaluation of the ability of the factor solution to represent the correlation structure, and (B) clinical and theoretical interpretability of the solution. A scree plot of the principal component solution was used as a guide to the number of factors that were needed.

#### Reliability

Internal Consistency was assessed by Cronbach’s alpha [33]. For scales or domains that are relatively unilateral, reliability was expected to be high (>0.80); for scales or domains that are more multifaceted, a lower reliability of at least 0.70 was considered acceptable.

Test-retest reliability was analyzed for those subjects who completed the re-test approximately 12 days after completing the COS. Given that the women had experienced both oocyte retrieval and embryo transplant in between administrations, it was expected that the re-test would not only reveal the degree of error variation that is a characteristic of the test, but would also be sensitive to changes in perception of the original COSI and thus, not be as strong a correlation as generally expected for test-retest reliability.

#### Validity

In order to assess convergent validity, hypotheses regarding the relationship between the measure assessing the similar concept to the COSI score were developed. Pearson’s correlations were computed to measure the association between the total and/or subscale scores on the COSI measure for which the significant relationship was expected.

In order to assess discriminate validity, hypotheses regarding the relationship between known groups and the COSI score being tested were developed. The scores of the groups on the COSI domains were compared using descriptive statistics, and parametric tests, typically one-way ANOVA with groups as a fixed factor (or a *t*-test in case of two groups and appropriate contrasts comparing groups when several groups were involved) to test for significance between the known groups and the COSI score. Table 1 presents the hypotheses for the tests of validity.

#### Exploratory analysis

The relationship between the COSI and other patient data (e.g. age, ethnicity, cause of infertility), key treatment factors (e.g. number of injections), and the women’s relationship to their fertility clinic (e.g. ease of contact) was examined using regression analysis for continuous variables and one way ANOVA for categorical variables.

## Results

### Sample description

The COSI validation sample consisted of 267 women (United Kingdom: 80: Ireland: 45; United States: 142) who completed the validation battery. The aggregate sample had a mean age of 35.7 years. 85.0% of the participants reported excellent or very good health. 79.4% of the sample was self-identified as White/Caucasian. The largest non-White/Caucasian group represented in the sample was Asian-American/Pacific Islander (7.9%) followed by mixed race (5.2%) or another ethnic group not listed (4.1%), Latino/Hispanic (1.9%), and Black/African American (1.5%). Additionally, a majority of the sample was employed 30 or greater hours per week (72.7%), and a majority earned equal to or greater than $50,000 annually (92.1%) with over half of the sample earning more than $100,000 annually (60.3%). 73.4% of the sample had no children. The full demographic and infertility factors description is shown in Table 2. On average there were no marked differences in socio-demographic factors across the three countries included.

**Table 2:** Demographic and infertility factors

	**Country**							
	**UK (N = 80)**		**Ireland (N = 45)**		**US (N = 142)**		**Total (N = 267)**	
**Age (years) at COSI date**								
N	79		45		141		265	
Mean (SD)	36.3	(4.0)	35.9	(3.7)	35.2	(4.7)	35.7	(4.4)
**General health, n (%)**								
Excellent/Very good	69	(86.3)	38	(84.4)	120	(84.5)	227	(85.0)
Good	10	(12.5)	7	(15.6)	21	(14.8)	38	(14.2)
Fair	1	(1.3)	0		0		1	(0.4)
Missing	0		0		1	(0.7)	1	(0.4)
**Employment status, n (%)**								
Employed - 30 or more hours/Week	57	(71.3)	30	(66.7)	107	(75.4)	194	(72.7)
Employed - less than 29 hours/Week	14	(17.5)	9	(20.0)	15	(10.6)	38	(14.2)
Unemployed (seeking employment)	3	(3.8)	2	(4.4)	3	(2.1)	8	(3.0)
Not seeking employment (homemaker, retired, etc.)	6	(7.5)	4	(8.9)	16	(11.3)	26	(9.7)
Missing	0		0		1	(0.7)	1	(0.4)
**Highest level of education, n (%)**								
Grade school (elementary) or less or Secondary school or technical school (some coursework or graduated)	7	(8.8)	12	(26.7)	9	(6.3)	28	(10.5)
College or further education or Higher education (completed graduation)	43	(53.8)	23	(51.1)	89	(62.7)	155	(58.1)
Post graduate studies	30	(37.5)	10	(22.2)	44	(31.0)	84	(31.5)
**Annual household income, n (%)**								
Less than £10 OR less than $19,900	1	(1.3)	0		2	(1.4)	3	(1.1)
£10 - £25,999 OR $20,000–$49,999	5	(6.3)	4	(8.9)	7	(4.9)	16	(6.0)
£25 - £50,999 OR $50,000–$99,999	26	(32.5)	14	(31.1)	45	(31.7)	85	(31.8)
More than £51,000 OR more than $100,000	47	(58.8)	27	(60.0)	87	(61.3)	161	(60.3)
Missing	1	(1.3)	0		1	(0.7)	2	(0.7)
**Number of other living children, n (%)**								
0	67	(83.8)	28	(62.2)	101	(71.1)	196	(73.4)
1 or more	12	(15.0)	14	(31.1)	40	(28.2)	66	(24.7)
Missing	1	(1.3)	3	(6.7)	1	(0.7)	5	(1.9)
**IVF treatment payment, n (%)**								
Self	59	(73.8)	41	(91.1)	56	(39.4)	156	(58.4)
Insurance/NHS	21	(26.3)	2	(4.4)	41	(28.9)	64	(24.0)
Combination self and insurance	0		1	(2.2)	40	(28.2)	41	(15.4)
Other	0		1	(2.2)	5	(3.5)	6	(2.2)
**Cause infertility, n (%)**								
Male factor	31	(38.8)	19	(42.2)	59	(41.5)	109	(40.8)
Tubal factor	15	(18.8)	4	(8.9)	21	(14.8)	40	(15.0)
Endometriosis	10	(12.5)	5	(11.1)	10	(7.0)	25	(9.4)
Cervical mucus problems	1	(1.3)	0		1	(0.7)	2	(0.7)
Unexplained infertility	23	(28.8)	15	(33.3)	28	(19.7)	66	(24.7)
Missing or unknown	0		2	(4.4)	23	(16.2)	25	(9.4)
**Duration of infertility, n (%)**								
<1 year	1	(1.3)	0		6	(4.2)	7	(2.6)
1- ≤2 years	14	(17.5)	11	(24.4)	55	(38.7)	80	(30.0)
2- ≤4 years	33	(41.3)	17	(37.8)	36	(25.4)	86	(32.2)
Over 4 years	27	(33.8)	14	(31.1)	32	(22.5)	73	(27.3)
Missing	5	(6.3)	3	(6.7)	13	(9.2)	21	(7.9)

*COSI* Controlled ovarian stimulation impact measure, *IVF* In vitro fertilization, *NHS* National health service, *PCOS* Polycystic ovarian syndrome, *AMA* Advanced maternal age.

### Item reduction and measurement model

Item reduction resulted in a final 28-item COSI. Four factors were identified: Interference in Daily Life (with 2 subdomains of Work and Home), Injection Burden, Psychological Health and Compliance Worry. These domains varied slightly from the a-priori hypothesized domains, which also included Side Effects and a separate domain for Work Interference, and did not include Injection Worry. As a result, the a-priori concurrent and known-groups validity hypotheses for the Side Effects domain were not tested, and post-hoc hypotheses for the new Compliance Worry were formulated based. A-priori hypotheses for the Work Interference domain were used to support the Work Interference subdomain of the Daily Life Interference domain. Incomplete data was minimal and did not affect item reduction.

The final factor structure of the core COSI items (excluding work items) is shown in Table 3.

**Table 3:** **Factor-analysis of core COSI items using a 4 factor solution and Varimax rotation**^**†**^

**Variable**	**Factor**				**Communality**
	**1**	**2**	**3**	**4**	
	**Psychological health**	**Compliance worry**	**Interference in daily life**	**Injection burden**	
Feel stressed	77			21	0.68
Feel emotional ups and downs	73				0.59
Feel emotionally exhausted	73		23		0.61
Feel anxious	66			21	0.51
Feel depressed	63		21		0.46
Feel strain or tension in relationship	54				0.32
Feel not able to manage life	49		30		0.35
Feel good about self*	47				0.29
Feel physically exhausted	47				0.26
Bothered: side effects from the med.	46				0.25
Worry: taking the correct dose		84			0.75
Worry: injecting properly		80		28	0.73
Worry: missing a dose		66	21		0.50
Worry: medication at the right time	27	64	21		0.54
Problem: learn how to take properly		45	30	27	0.38
Problem: manage or schedule time	25	21	85		0.84
Problem: keep up family/soc. life	35		62		0.53
Problem: travel or short trips			58		0.38
Convenient take meds at corr. time*			40		0.21
Problem: schedule monitoring visits		24	39		0.25
Bothered: injections	22		21	89	0.91
Bothered: injections required	29			76	0.72
Bothered: having to inject myself		28		60	0.45
Bothered: problems injection site	32		21	43	0.34

† Excluding work-related questions, including imputed missing values.

* Variables have been mirrored for the analysis. Factor loadings have been multiplied by 100.

Factor loadings with an (absolute) value <0.2 were not printed.

The communality indicates the proportion of variance explained by the 4 factor solution.

*COSI* Controlled ovarian stimulation impact measure.

### Reliability

Cronbach Alpha’s for the internal consistency of all domains, and the total score were between 0.80 to 0.92 thus meeting the criteria of acceptable internal consistency. Test–retest correlations were also acceptable (between 0.72 to 0.87) for all domains and the total score. Reliability ICC statistics on the COSI are shown in Table 4.

**Table 4:** Reliability ICC statistics on the COSI

**Scale identification**	Reliability*	**Test-retest reliability**
COSI Total	0.92	0.87
Interference in Daily Life		
Work	0.80	0.80
Home	0.80	0.72
Compliance Worry	0.84	0.78
Psychological Health	0.87	0.82
Injection Burden	0.84	0.83

*Reliability based on internal consistency using Cronbach’s alpha.

*COSI* Controlled ovarian stimulation impact measure.

### Validity

All a-priori hypotheses for convergent and discriminant validity were met. Additionally, the post-hoc convergent validity hypotheses for the Injection Burden domain were also met.

### Final measure

The final COSI consists of 28 items in 4 domains (Interference in Daily Life: Home 5 items, Work 4 items; Psychological Health: 10 items; Injection Burden: 4 items; Compliance Worry: 5 items).

**Figure 1:**
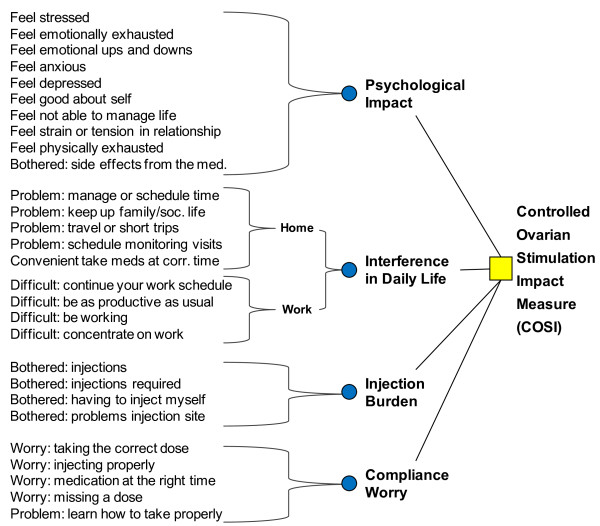
Conceptual framework.

The conceptual framework of items per domain of the COSI is as shown in Figure 1.

### Exploratory analyses

There was no significant relationship between COSI Total score or any domains and major demographic factors: age, marital status, ethnicity (with exception of Injection Burden: p = 0.007, higher impact for Asian and mixed race), employment status, education, treatment protocol (with exception of Interference in Daily Life - Work: p = 0.019, lower impact for agonist and antagonist short flare-up protocols), income (with exception of Interference in Daily Life: p = 0.024, higher impact for income above $100.000) or previous injection experience. The overall impact of COS was lower (COSI total score: p = 0.025) as well as the Interference in Daily Life (home: p = 0.005 work p = 0.037) and Injection Burden (p = 0.031) for those who were paying either all or some of the costs of IVF themselves.

With regard to treatment factors, women who felt they were better trained to self administer injections also had lower overall impact (p = 0.002) as well as lower Interference in Daily Life (home: p = 0.002, work: p = 0.05) and Injection Burden (p = 0.032). Also, women who injected themselves reported lower Injection Burden then when this was done by the husband or shared (p = 0.002). Further, women reported lower overall impact (p = 0.013), Psychological Impact (p = 0.047), Interference in Daily Life at home (p = 0.04), and less Injection Burden (p = 0.017) when the cause of infertility was related to a male factor. Women who had a greater number of injections had greater Injection Burden (p = 0.009), and depending on the number of monitoring visits (categorized as ≤3, 4–6, 7–9, ≥10) did not experience equal Overall Impact (p < 0.001), Psychological Impact (p = 0.002), Interference in Daily Home Life (p = 0.044), and Compliance Worry (p = 0.044), with categories corresponding to lower number of visits corresponding to improved scores.

The relationship the women had with their fertility clinic also played a role on the impact of treatment. Women who felt they had a better relationship with their clinics, or that the clinics were easy to contact and had fewer monitoring visits, had significantly better outcomes in both overall impact as well as for most domains. Additionally, significant relationships (p < 0.001) were found between the COSI Total Score and for how stressful women felt the infertility treatment was, the degree of supportiveness of spouse and friends and family, their need to rearrange work schedule and the degree of supportiveness and ease of contacting their fertility clinic. Regarding the Daily Life Interference domain_,_ women who worked, and who needed to rearrange their work schedule due to their COS treatment, did have greater Daily Life Work Interference (p < 0.001), and women with a greater number of monitoring visits had greater Daily Life Home Interference (p < 0.001) and Daily Life Work Interference if they worked (p = 0.003).

## Discussion

These findings suggest that the 28-item COSI can be considered a well-developed and valid PRO measure with well-defined domains (Interference in Daily Life (with 2 subdomains of Work and Home), Injection Burden, Psychological Health and Compliance Worry) which assess the impact of COS on women’s functioning and well-being. Further, each of these domains has been found to be acceptable psychometrically and can be considered appropriate for use as individual concepts. IVF has been found to create anxiety in patients [21] and this anxiety may be related to treatment outcomes [15-19]. Further, discontinuation of fertility treatment has been attributed to the burden of treatment [34]. It has been suggested that further evaluation of the efficacy of treatments and interventions that reduce burden is needed [34]. The COSI has been rigorously developed following the Guidance for industry: patient-reported outcome measures [22], as a measure of the impact of COS on patient functioning and well-being. This study has shown that the COSI is relevant to patients, clinically meaningful, valid and reliable. As such, the COSI should be helpful to clinicians to assess the multiple impacts of COS and the effectiveness of interventions geared to reduce this anxiety.

The impact of a treatment can often be mitigated by many factors such as patient characteristics, level of family support, health process factors and characteristics of the treatment [21,35,36]. In this study, we also found, as shown in the exploratory analyses that the impact of COS on women is not insignificant and that this impact can be mitigated by factors such as where the procedure is conducted, the type of treatment, number of visits and injections as well as family, clinic and work support structures. A greater focus on these factors by clinicians should help to improve care and reduce the burden of infertility.

Some limitations and future considerations should be mentioned. Unfortunately, due to ethics requirements, we were not able to collect any information on women who choose not to participate in the validation study, thus the extent to which women who did participate were similar to women who did not participate is unknown. However, the characteristics of the women who did participate appear to be representative of the general population of women undergoing COS. Further, the study was conducted in Western countries and the sample was limited in ethnic diversity. Although the exploratory analyses did not find an effect of ethnicity on the impact of COS, future research is needed to confirm this finding. The exploratory analyses also suggested that site characteristics may impact outcomes and this also should be an area for future research in order to more clearly define site characteristics that help rather than hinder women in coping with COS. Finally, as this was a validation study, translation issues have not yet been addressed. It will be important that any translations of the COSI take into consideration cultural equivalency in the translation process.

Validation is an iterative process and should be continually ongoing as PRO measures are initially developed and used in clinical practice. Further work on the COSI should examine responsiveness and interpretability of the COSI scores in future studies. Further, as this study was conducted in western cultures, additional validation work in non-western cultures would be warranted.

## Conclusions

The study findings suggest that the 28-item COSI can be considered a well-developed and valid PRO measure with well-defined domains which assess the impact of COS on women’s functioning and well-being.

## Abbreviations

COS: Controlled ovarian stimulation; COSI: Controlled ovarian stimulation impact measure; hCG: Human chorionic gonadotrophin; ICSI: Intracytoplasmic sperm injection; IVF: In vitro fertilization; OBLIMIN: oblique rotation; PRO: Patient reported outcome measure.

## Competing interests

Dr. Brod is a paid advisor/paid consultant to Merck & Co., Inc. Dr. Fennema is an employee of Merck Sharp & Dohme BV.

## Authors’ contributions

MB and HF contributed to the design, analysis, data interpretation and manuscript preparation. Both authors read and approved the final manuscript.

## References

[B1] Verhaak CM Smeenk JM Evers AW Kremer JA Kraaimaat FW Braat DD Women's emotional adjustment to IVF: a systematic review of 25 years of research Hum Reprod Update 2007 13 27 36 16940360 10.1093/humupd/dml040

[B2] Hynes GJ Callan VJ Terry DJ Gallois C The psychological well-being of infertile women after a failed IVF attempt: the effects of coping Br J Med Psychol 1992 65 Pt 3 269 278 1390361 10.1111/j.2044-8341.1992.tb01707.x

[B3] Slade P Emery J Lieberman BA A prospective, longitudinal study of emotions and relationships in in-vitro fertilization treatment Hum Reprod 1997 12 183 190 10.1093/humrep/12.1.183 9043926

[B4] Visser AP Haan G Zalmstra H Wouters I Psychosocial aspects of in vitro fertilization J Psychosom Obstet Gynaecol 1994 15 35 43 10.3109/01674829409025627 8038887

[B5] Beaurepaire J Jones M Thiering P Saunders D Tennant C Psychosocial adjustment to infertility and its treatment: male and female responses at different stages of IVF/ET treatment J Psychosom Res 1994 38 229 240 10.1016/0022-3999(94)90118-X 8027962

[B6] Salvatore P Gariboldi S Offidani A Coppola F Amore M Maggini C Psychopathology, personality, and marital relationship in patients undergoing in vitro fertilization procedures Fertil Steril 2001 75 1119 1125 10.1016/S0015-0282(01)01775-7 11384636

[B7] Ardenti R Campari C Agazzi L La Sala GB Anxiety and perceptive functioning of infertile women during in-vitro fertilization: exploratory survey of an Italian sample Hum Reprod 1999 14 3126 3132 10.1093/humrep/14.12.3126 10601108

[B8] Klonoff-Cohen H Chu E Natarajan L Sieber W A prospective study of stress among women undergoing in vitro fertilization or gamete intrafallopian transfer Fertil Steril 2001 76 675 687 10.1016/S0015-0282(01)02008-8 11591398

[B9] Yong P Martin C Thong J A comparison of psychological functioning in women at different stages of in vitro fertilization treatment using the mean affect adjective check list J Assist Reprod Genet 2000 17 553 556 10.1023/A:1026429712794 11209535 PMC3455452

[B10] Merari D Feldberg D Elizur A Goldman J Modan B Psychological and hormonal changes in the course of in vitro fertilization J Assist Reprod Genet 1992 9 161 169 10.1007/BF01203757 1627933

[B11] Boivin J Takefman JE Impact of the in-vitro fertilization process on emotional, physical and relational variables Hum Reprod 1996 11 903 907 10.1093/oxfordjournals.humrep.a019276 8671350

[B12] Boivin J Takefman JE Stress level across stages of in vitro fertilization in subsequently pregnant and nonpregnant women Fertil Steril 1995 64 802 810 7672154 10.1016/s0015-0282(16)57858-3

[B13] Verhaak CM Smeenk JM Eugster A van Minnen A Kremer JA Kraaimaat FW Stress and marital satisfaction among women before and after their first cycle of in vitro fertilization and intracytoplasmic sperm injection Fertil Steril 2001 76 525 531 10.1016/S0015-0282(01)01931-8 11532476

[B14] Lok IH Lee DT Cheung LP Chung WS Lo WK Haines CJ Psychiatric morbidity amongst infertile Chinese women undergoing treatment with assisted reproductive technology and the impact of treatment failure Gynecol Obstet Invest 2002 53 195 199 10.1159/000064560 12186982

[B15] Gourounti K Anagnostopoulos F Vaslamatzis G The relation of psychological stress to pregnancy outcome among women undergoing in-vitro fertilization and intracytoplasmic sperm injection Women Health 2011 51 321 339 10.1080/03630242.2011.574791 21707337

[B16] Smeenk JM Verhaak CM Vingerhoets AJ Sweep CG Merkus JM Willemsen SJ van Minnen A Straatman H Braat DD Stress and outcome success in IVF: the role of self-reports and endocrine variables Hum Reprod 2005 20 991 996 15665011 10.1093/humrep/deh739

[B17] Campagne DM Should fertilization treatment start with reducing stress? Hum Reprod 2006 21 1651 1658 10.1093/humrep/del078 16543257

[B18] Eugster A Vingerhoets AJ Psychological aspects of in vitro fertilization: a review Soc Sci Med 1999 48 575 589 10.1016/S0277-9536(98)00386-4 10080360

[B19] Csemiczky G Landgren BM Collins A The influence of stress and state anxiety on the outcome of IVF-treatment: psychological and endocrinological assessment of Swedish women entering IVF-treatment Acta Obstet Gynecol Scand 2000 79 113 118 10.1034/j.1600-0412.2000.079002113.x 10696958

[B20] Anderheim L Holter H Bergh C Moller A Does psychological stress affect the outcome of in vitro fertilization? Hum Reprod 2005 20 2969 2975 10.1093/humrep/dei219 16123098

[B21] Brod M Verhaak CM Wiebinga CJ Gerris J Hoomans EH Improving clinical understanding of the effect of ovarian stimulation on women's lives Reprod Biomed Online 2009 18 391 400 10.1016/S1472-6483(10)60098-3 19298739

[B22] U.S. Dept. of Health and Human Services, Food and Drug Administration, Center for Drug Evaluation and Research: Center for Biologics Evaluation and Research: Center for Devices and Radiological Health Guidance for industry: patient-reported outcome measures, use in medical product development to support labeling claims [http://www.fda.gov/downloads/Drugs/GuidanceComplianceRegulatoryInformation/Guidances/UCM193282.pdf]

[B23] Juniper E Guyatt G Jaeschke R Spilker B How to develop and validate a new health-related quality of life instrument Quality of life and pharmacoeconomics in clinical trials 1996 2 Philadelphia: Lippincott-Raven 49 56

[B24] The Netherlands Cancer Institute, Amsterdam Assessing health status and quality-of-life instruments: attributes and review criteria Qual Life Res 2002 11 193 205 10.1023/A:1015291021312 12074258

[B25] Snoek FJ Mollema ED Heine RJ Bouter LM van der Ploeg HM Development and validation of the diabetes fear of injecting and self-testing questionnaire (D-FISQ): first findings Diabet Med 1997 14 871 876 10.1002/(SICI)1096-9136(199710)14:10<871::AID-DIA457>3.0.CO;2-Y 9371481

[B26] Endicott J Nee J Harrison W Blumenthal R Quality of life enjoyment and satisfaction questionnaire: a new measure Psychopharmacol Bull 1993 29 321 326 8290681

[B27] Endicott J Nee J Endicott Work Productivity Scale (EWPS): a new measure to assess treatment effects Psychopharmacol Bull 1997 33 13 16 9133746

[B28] Wisniewski SR Rush AJ Balasubramani GK Trivedi MH Nierenberg AA Self-rated global measure of the frequency, intensity, and burden of side effects J Psychiatr Pract 2006 12 71 79 10.1097/00131746-200603000-00002 16728903

[B29] Carpenter JS The hot flash related daily interference scale: a tool for assessing the impact of hot flashes on quality of life following breast cancer J Pain Symptom Manage 2001 22 979 989 10.1016/S0885-3924(01)00353-0 11738160

[B30] Anderson RT Skovlund SE Marrero D Levine DW Meadows K Brod M Balkrishnan R Development and validation of the insulin treatment satisfaction questionnaire Clin Ther 2004 26 565 578 10.1016/S0149-2918(04)90059-8 15189754

[B31] Atkinson MJ Sinha A Hass SL Colman SS Kumar RN Brod M Rowland CR Validation of a general measure of treatment satisfaction, the Treatment Satisfaction Questionnaire for Medication (TSQM), using a national panel study of chronic disease Health Qual Life Outcomes 2004 2 12 10.1186/1477-7525-2-12 14987333 PMC398419

[B32] Dupuy H Wender N, Mattson M, Furburg C, Elinson J The psychological general well-being (PGWB) index Assessment of quality of life in clinical trials of cardiovascular therapies 1984 New York: Le Jacq 170 183 10.1016/s0002-9149(84)80232-5 6333175

[B33] Chronbach LJ Coefficient alpha and the internal structure of tests Psychometrika 1951 16 297 334 10.1007/BF02310555

[B34] Boivin J Domar AD Shapiro DB Wischmann TH Fauser BC Verhaak C Tackling burden in ART: an integrated approach for medical staff Hum Reprod 2012 27 941 950 10.1093/humrep/der467 22258661

[B35] Bleil ME Pasch LA Gregorich SE Millstein SG Katz PP Adler NE Fertility treatment response: is it better to be more optimistic or less pessimistic? Psychosom Med 2012 74 193 199 10.1097/PSY.0b013e318242096b 22286845 PMC3273547

[B36] Verhaak CM Smeenk JM van Minnen A Kremer JA Kraaimaat FW A longitudinal, prospective study on emotional adjustment before, during and after consecutive fertility treatment cycles Hum Reprod 2005 20 2253 2260 10.1093/humrep/dei015 15817584

